# Adaptation of the Serious Illness Conversation Guide to Singapore's Multicultural Setting for Patients With Heart Failure, Renal Failure, or Cancer

**DOI:** 10.1089/pmr.2023.0086

**Published:** 2024-03-26

**Authors:** Anna So Youn Lee, Swee Noi Tang, Gillian Li Gek Phua, Alethea Chung-Pheng Yee, Shirlyn Hui-Shan Neo

**Affiliations:** ^1^ Division of Supportive and Palliative Care, National Cancer Centre Singapore, Singapore, Singapore.; ^2^ Office of Research, Duke-NUS Medical School, Lien Centre for Palliative Care, Singapore, Singapore.

**Keywords:** community-based palliative care, cross-cultural issues, Asian populations, health services research, hospital-specific palliative care issues, Serious Illness Conversation Guide

## Abstract

**Background:**

The Serious Illness Conversation Guide (SICG) was developed by Ariadne Labs in the United States. However, there is a scarcity of literature on the cross-cultural adaptations of the SICG in Asian settings.

**Objectives:**

We aimed to adapt the SICG for English-speaking patients with serious illnesses in Singapore.

**Methods:**

We purposively recruited 28 patients with advanced stages of heart failure, renal failure, or cancer from a tertiary hospital. A designated research team member conducted semistructured interviews to obtain participants' feedback on the SICG. The interviews were transcribed by the designated study team member. Participants' response to each item on the SICG was coded quantitatively into categories to denote participant acceptance, partial acceptance, or nonacceptance. Transcripts were further analyzed using content analysis to understand participants' rationale regarding feedback of the specific SICG item. Modifications to the SICG were iteratively made over time to obtain its current version.

**Results:**

Participants indicated a preference for direct language with shorter sentences and inclusive pronouns. It was considered important that clinicians keep the conversation hopeful, individualize the conversation content according to the patient's journey, and use prompts where necessary to support the patient's elaboration.

**Conclusion:**

This study outlined a patient-centric approach to localizing the SICG in the English language to a new cultural context, marking the first such effort in an Asian setting. Further study is under way to evaluate the SICG in more disease populations and non-English languages used in Singapore.

## Introduction

The Serious Illness Conversation Guide (SICG) is a valuable and structured communication tool, developed by Ariadne Labs in the United States.^1^ However, the efficacy of such tools depends on the clinicians' ability to elicit the patient's values in a culturally appropriate manner.^2^ Therefore, it is crucial to ensure that the words used in the SICG foster cultural safety.^3^

Successful cultural adaptations have been undertaken primarily for its use within underserved, indigenous, or minority communities in North America and Europe.^4–7^ However, there is a gap in the literature on the topic of cross-continent application of the SICG in a multiethnic urban Asian population, such as Singapore, where differing attitudes toward end-of-life (EOL) decision making and care planning exist.^8–10^

According to Singapore's Consensus of Population 2020 Report, English was the predominant language spoken at home by 48.3% of residents aged five years and above.^11^ Therefore, our study team first aimed to adapt the US version of the SICG by Ariadne Labs to the English language used in Singapore.

## Methods

### Settings and participants

The participants in this study were patients diagnosed with one or more serious illnesses as defined by our inclusion criteria (Table 1). The inclusion criteria reflect the top three prevalent life-limiting illnesses in Singapore.^12^

**Table 1.** Sampling and Inclusion/Exclusion Criteria

Inclusion criteria
For patient participants, sampling will ensure diversity across age, race, educational status and gender. Specific disease criteria for inclusion include:
1. Advanced solid tumor cancers or advanced hematological cancers (stage 3 or 4)
2. Advanced renal failure (defined as having a creatinine clearance of <15 mL/min)
3. Advanced heart failure (Stage C and Stage D; defined by the American Heart Association/American College of Cardiology staging)
Exclusion criteria
Participants who cannot sign informed consent will be excluded.

The sample was recruited purposively across demographic factors to obtain diverse perspectives as displayed in Table 1.

### Data collection

A designated research team member (A.S.Y.L.) screened the outpatient clinic lists to determine whether they fulfilled the inclusion criteria. Eligible patients were recruited for this study. Recruitment was conducted at three study sites providing care for a large number of patients with serious illnesses. These sites were Singapore General Hospital (SGH), National Heart Centre Singapore (NHCS), and National Cancer Centre Singapore (NCCS). Essential sociodemographic and medical diagnostic information using screening data from electronic medical records was gathered for each participant.

Before the commencement of the research project, the interviewer (A.S.Y.L.) underwent training in consent procedures and semistructured interview techniques. She further familiarized herself with the SIC program through guidance from the staff affiliated with Ariadne Labs.

Semistructured individual interviews were conducted in English and held either in person or through a Zoom videoconference. Interviews lasted up to 60 minutes and were structured into three parts: first, review of the SICG sentence by sentence to gather specific feedback, regarding its clarity, language, flow, and structure; second, open-ended questions exploring patients' past and/or current and preferred communication styles with their health care professionals; third, discussion of the barriers and enablers related to communication about serious illnesses and EOL care, guided by the Reach, Effectiveness, Adoption, Implementation, and Maintenance (RE-AIM) Planning and Evaluation framework and the Capacity, Opportunity, and Motivation model for Behavioral change (COM-B) (Supplementary Appendix SA1).

Concerning the first aspect of SICG feedback, the interviewer (A.S.Y.L.) asked participants to provide feedback through a color-coding scheme (Supplementary Appendix SA2), which followed a logic similar to that of traffic lights.^2^ If patients suggested changes (orange) or indicated omissions (red) in any part of the SICG, the interviewer paused to inquire about a more detailed thought process (“Which part of the sentence does not sit well with you, and how would you change the phrase?”).

Overall, the interviewer ensured that participants had enough time to “read,” “think,” and “comprehend” the SICG content and wording during the interview in accordance with other studies that culturally adapted the SICG to their local language.^2,4,6^ If participants had queries about the SICG and interview process, adequate time was provided to address their questions and ensure comprehension.

### Data analysis

All interviews were recorded and transcribed word-by-word for data analysis. Participant's response to each item on the SICG was coded quantitatively into categories (red, orange, or green) to denote participant acceptance, partial acceptance, or nonacceptance. The interview data were further analyzed using content analysis^13^ to understand participants' rationale for level of acceptance of the specific SICG item.

To allow for an iterative review of the SICG over time, the interviewer (A.S.Y.L.) convened with the Principal Investigator (PI) (S.N.T.) after every set of five to six interviews. These meetings served as a forum to consolidate and integrate the findings, thereby facilitating the incorporation of linguistic changes into the SICG for subsequent testing. The color-coded data from each interview set was compiled into an Excel file (Supplementary Appendix SA3), and feedback saturation was defined as predominantly obtaining “green” responses by the end of the study.

To ensure the validity of the final local version of the SICG, the interviewer (A.S.Y.L.) contacted all 28 participants to ensure acceptability of the final version of the SICG. In addition, the final version was reviewed by experts in palliative care in Singapore and by an affiliated member of Ariadne Labs to ensure face validity.

### Ethics

This study obtained approval from the institutional review board at the SingHealth Centralized Institutional Review Board (CIRB; Reference number: 2022/2736).

## Results

### The final sample comprised 28 patients

For the first round of interviews, we recruited five interviewees with advanced cancer. Participants mainly gave feedback emphasizing the importance of (1) using a hopeful language, (2) incorporating inclusive first-person pronouns (e.g., “we,” “us,” and “ours”) to foster a sense of teamwork, and (3) avoiding ominous words and phrases such as “but” and “I am worried.” In addition, participants expressed a preference for a more direct language with shorter sentences.

In the second round of interviews, we subsequently further recruited six patients with advanced cancer to confirm the findings from the first five patients with advanced cancer. In this round, the participants similarly voted for simpler and shorter phrases. Participants expressed a preference for prompts, examples, and follow-up questions to assist them in formulating responses during conversations about serious illnesses. For instance, they unanimously found the examples listed under the “trade-offs” question (Supplementary Appendix SA4) to be beneficial. In addition, participants recommended creating two versions of certain question stems such as splitting the “critical abilities” question into “early-” and “late-trajectories.”

Finally, in the last round of interviews, we recruited five additional participants with advanced cancer to review the SICG. No further changes were suggested. We then proceed to recruit 12 noncancer participants, comprising 6 with advanced heart failure and 6 with advanced renal failure. Notably, feedback from noncancer participants was consistent with the prior suggestion by advanced cancer participants. They also suggested using straightforward language particularly regarding the sentence under the “Share” section of the SICG.

To confirm the acceptability of the final version of the SICG, the study team managed to contact 14 out of 28 participants for a second review. These 14 patients confirmed its acceptability. Regarding the remaining 14 patients, 11 were unreachable, 1 declined, and 2 had passed away. Experts in serious illness communication, both within our local clinical setting and in the United States, verified the acceptability of our final SICG.

## Discussion

A patient-centric approach was utilized in this study to guide the methodology for interviews and data collection, thereby facilitating a seamless localization of the SICG to the specific cultural context of Singapore. Our approach integrated patient feedback through color-coded visualization and the inclusion of patient-suggested English expressions, while adhering to a systematic refinement process. We iteratively reviewed and adjusted the SICG after every five to six participant interviews to incorporate their suggestions without compromising our team's focus.

During the interviews, we found that certain aspects of our data aligned with changes observed in the latest US-tested version published by Ariadne Labs in May 2023.^14^ Similar to their counterparts in the United States, our participants in Singapore expressed that it was important that clinicians use uplifting language, whether through hopeful phrasing, plural first-person pronouns, or avoidance of negative words and phrases.

Interestingly, our team encountered cultural nuances that diverged from those of their Western counterparts. Surface-level observations revealed participants' strong preferences for direct succinct wording and patient-targeted or patient-specific questions, which reflect Singapore's dialectical use of English. These findings could be attributed to preferences that are specific to Singapore's multiethnic Asian cultures, where patients might expect clinicians to strictly adhere to matters related to medical treatment and avoid discussing matters related to taboo topics such as death.^7^

The strength of this study is that it validated the SICG multidimensionally through patient interviews, local palliative care experts, and with a staff affiliated to Ariadne Labs. Our team's systematic approach to uncovering cultural nuances for the localization of a well-known communication tool further adds a novel dimension.

## Conclusions

Our study team developed a culturally adapted tool that can be used in Singapore's multicultural society (Supplementary Appendix SA4). This report further highlights the importance of a patient-centered approach in research and ensuring cultural safety and appropriateness before large-scale implementation of the SICG. Our team plans to expand the present study to evaluate the SICG in other languages common in Singapore such as Mandarin, as well as test the SICG in other illnesses, such as chronic neurological disorders, dementia, and frailty.

## Supplementary Material

Supplemental data

Supplemental data

Supplemental data

Supplemental data

## Abbreviations Used

EOL: end-of-life
NCCS: National Cancer Centre Singapore
NHCS: National Heart Centre Singapore
SGH: Singapore General Hospital
SICG: Serious Illness Conversation Guide
