# Supplementary material for: Adaptation of the Serious Illness Conversation Guide to Singapore's Multicultural Setting for Patients With Heart Failure, Renal Failure, or Cancer
Source: Palliat Med Rep. 2024 Mar 26;5(1):122–6. doi: 10.1089/pmr.2023.0086 (PMC10979657; doi:10.1089/pmr.2023.0086)
Supplement: Supplemental data [file Suppl_AppendixS1.docx]

**Appendix A: Interviewing Guide (Patient)**

| REAIM/COM-B – Patient only  Would you be personally interested in engaging in such conversations with your healthcare staff?  What would help you to start engaging in such conversations?  What would make it difficult for you to engage in such conversations?  What do you think are suitable times and settings your healthcare team can talk about these topics with you?  Who in your healthcare team would you say, is most suitable to start and continue this conversation with you?  What is the best way to keep these conversations going?  How do you think we can best prepare you before-hand, to have these conversations?  What is the best way for healthcare staff to reach out to other patients like yourself to have these conversations? |
| --- |

Abbreviations Used:

REAIM: Reach, Effectiveness, Adoption, Implementation, and Maintenance

COM-B: Capacity, Opportunity, and Motivation model for Behavioral change
