# Supplementary material for: Adaptation of the Serious Illness Conversation Guide to Singapore's Multicultural Setting for Patients With Heart Failure, Renal Failure, or Cancer
Source: Palliat Med Rep. 2024 Mar 26;5(1):122–6. doi: 10.1089/pmr.2023.0086 (PMC10979657; doi:10.1089/pmr.2023.0086)
Supplement: Supplemental data [file Suppl_AppendixS3.docx]

**Appendix C: "Summary of findings" Excel – Blank**

| Version 1 | **Q#.#** | **Suggestion/ Elaboration** | **Q#.#** | **Suggestion/ Elaboration** | **Q#.#** | **Suggestion/ Elaboration** |
| --- | --- | --- | --- | --- | --- | --- |
| PT## | [sample] |  |  |  |  |  |
| PT## | [sample] |  |  |  |  |  |
|  |  |  |  |  |  |  |
| Version 2 | **Q#.#)** | **Suggestion/ Elaboration** | **Q#.#** | **Suggestion/ Elaboration** | **Q#.#** | **Suggestion/ Elaboration** |
| PT## |  |  |  |  |  |  |
| PT## |  |  |  |  |  |  |
|  |  |  |  |  |  |  |
| Version 3 | **Q#.#)** | **Suggestion/ Elaboration** | **Q#.#** | **Suggestion/ Elaboration** | **Q#.#** | **Suggestion/ Elaboration** |
| PT## |  |  |  |  |  |  |
| PT## |  |  |  |  |  |  |
