# Supplementary material for: Adaptation of the Serious Illness Conversation Guide to Singapore's Multicultural Setting for Patients With Heart Failure, Renal Failure, or Cancer
Source: Palliat Med Rep. 2024 Mar 26;5(1):122–6. doi: 10.1089/pmr.2023.0086 (PMC10979657; doi:10.1089/pmr.2023.0086)
Supplement: Supplemental data [file Suppl_AppendixS4.docx]

**Appendix D: Locally patient-tested SICG**
